# Enzymes: An integrated view of structure, dynamics and function

**DOI:** 10.1186/1475-2859-5-2

**Published:** 2006-01-12

**Authors:** Pratul K Agarwal

**Affiliations:** 1 Computational Biology Institute, and Computer Science and Mathematics Division, Oak Ridge National Laboratory, Oak Ridge, Tennessee 37831, USA

## Abstract

Microbes utilize enzymes to perform a variety of functions. Enzymes are biocatalysts working as highly efficient machines at the molecular level. In the past, enzymes have been viewed as static entities and their function has been explained on the basis of direct structural interactions between the enzyme and the substrate. A variety of experimental and computational techniques, however, continue to reveal that proteins are dynamically active machines, with various parts exhibiting internal motions at a wide range of time-scales. Increasing evidence also indicates that these internal protein motions play a role in promoting protein function such as enzyme catalysis. Moreover, the thermodynamical fluctuations of the solvent, surrounding the protein, have an impact on internal protein motions and, therefore, on enzyme function. In this review, we describe recent biochemical and theoretical investigations of internal protein dynamics linked to enzyme catalysis. In the enzyme cyclophilin A, investigations have lead to the discovery of a network of protein vibrations promoting catalysis. Cyclophilin A catalyzes peptidyl-prolyl *cis/trans *isomerization in a variety of peptide and protein substrates. Recent studies of cyclophilin A are discussed in detail and other enzymes (dihydrofolate reductase and liver alcohol dehydrogenase) where similar discoveries have been reported are also briefly discussed. The detailed characterization of the discovered networks indicates that protein dynamics plays a role in rate-enhancement achieved by enzymes. An integrated view of enzyme structure, dynamics and function have wide implications in understanding allosteric and co-operative effects, as well as protein engineering of more efficient enzymes and novel drug design.

## Introduction

**Figure 1:**
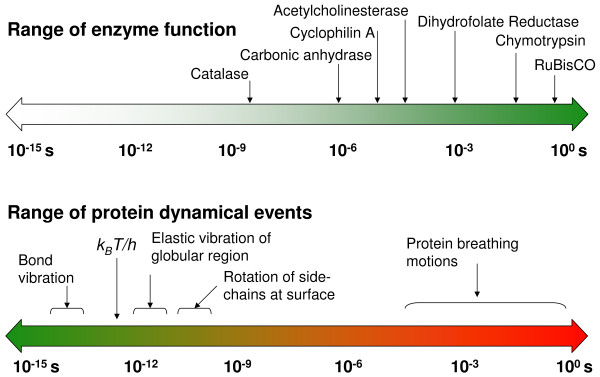
The range of time-scales involved in substrate turnover step of enzyme catalyzed reactions and internal protein dynamics are similar. Note the universal frequency factor (k_B_T/h), which is commonly used in transition state theory; *k*_*B *_is the Boltzmann's constant, *T *represents the ambient temperature and *h *is the Planck's constant.

Microbial cell factories operate as a collection of efficient molecular machines. The success of these factories depends on the efficiency of a particular class of biomolecules – protein enzymes. Enzymes are responsible for catalyzing reactions in a variety of biological processes in all living cells. It is well known that enzymes are highly efficient catalysts as they can accelerate reactions by as many as 17 orders of magnitude [1,2]. The factors that enable enzymes to provide the large enhancement of reaction rates; however, still remain a matter of discussion [3,4]. For more than a century, the activity of enzymes has been related to their structure; the "lock-and-key" and "induced-fit" hypotheses have suggested that the structural interactions between enzymes and the substrates play a role in enzyme catalysis [5,6]. Such a view is incomplete as it fails to explain allosteric and cooperative effects, as well as the detailed mechanism of the large rate-enhancement achieved by enzymes. Enzymes catalyze reactions on a wide range of time-scales, which are similar to the time-scales for various events of internal protein dynamics, raising the question whether dynamics and enzyme catalysis are interrelated or not (see Figure 1) [7-12]. It is known that protein dynamics plays a role in many aspects of enzyme function, including substrate/cofactor binding or release. Its connection to the substrate turnover step, however, has been challenging to ascertain.

An integrated view of protein structure, dynamics, and function is emerging, where proteins are considered as dynamically active machines and internal protein motions are closely linked to function such as enzyme catalysis. Currently there is wide interest, both from experimental and computational groups, in investigating this interconnection. A number of investigations have provided fascinating details about the movement of protein parts and their involvement in enzyme function. Techniques such as X-ray crystallography and small-angle scattering [13,14], NMR studies [15-17], hydrogen-deuterium exchange [18], neutron scattering [19], biochemical and mutational analysis [7,20,21] have provided vital clues at individual time-scales; however, the detailed understanding of protein dynamics requires information over a broad range of time-scales. Moreover, the hydration-shell and bulk solvent fluctuations have been suggested to impact protein dynamics, and therefore, protein function [22,23]. Theoretical studies and computational modeling are playing a vital role in investigating the link between protein dynamics, solvent fluctuations and enzyme catalysis at multiple time-scales [8,10-12].

In this review, we describe recent biochemical and theoretical/computational studies that have investigated the link between protein dynamics and enzyme catalysis. In particular, we describe the recent investigations of the peptidyl-prolyl *cis/trans *isomerization activity of the enzyme cyclophilin A, followed by a discussion on similar evidence from other enzyme reactions, namely the hydride transfer reactions catalyzed by dihydrofolate reductase and by liver alcohol dehydrogenase. There are wide implications of understanding the interconnection between protein structure, dynamics and function such as enzyme catalysis. It is known that enzymes catalyzing the same reactions belong to a protein "fold" family, where the overall characteristic shape of the protein is similar. Also, enzymes catalyzing mechanistically similar reactions often belong to the same super-family of protein fold. The benefits of better understanding of enzyme "folds" and dynamics include the possibility of improving the efficiency of microbial factories by engineering of enzymes, as well as designing new enzymes with novel functionalities. Further, there are medical implications of allosteric and cooperative effects for enzyme activity in novel drug design.

### Cyclophilin A

**Figure 2:**
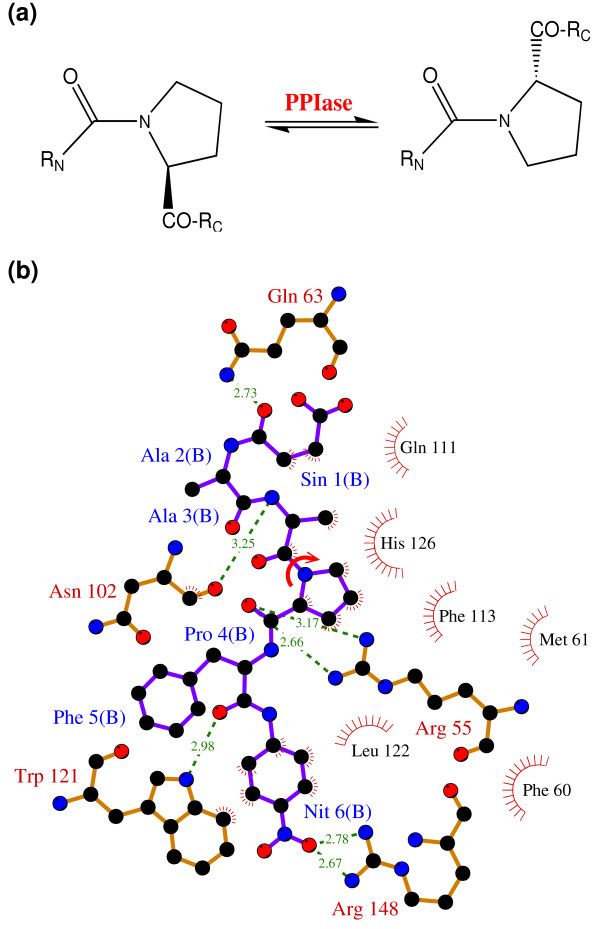
The reaction catalyzed by CypA (a) CypA is a member of a family of enzymes known as PPIase, which catalyze the *cis/trans *isomerization of peptide bonds N-terminal to proline residues in peptides and proteins (b) The active-site of CypA with a peptide substrate. The shown substrate has the sequence succinyl(Sin)-Ala-Ala-Pro-Phe-*p*-nitroanilide(Nit) and is labeled as chain B. The red arrow indicates the catalyzed isomerization. Several residues are conserved for their role in enzyme reaction. The dynamical motion of these hydrophobic and hydrophilic residues is linked to the substrate turnover step [10–12]. The green lines indicate hydrogen bonds between substrate and enzyme, while the hydrophobic interactions are depicted by small red radiating lines.

**Figure 3:**
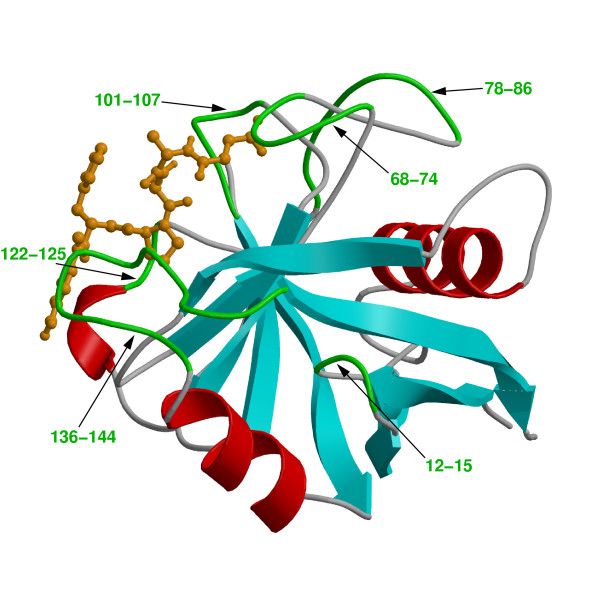
Three-dimensional structure of CypA. Protein secondary structure is represented with cyan arrows (β-sheets) and red helices (α-helices) based on crystal structure from Zhao and Ke (PDB code: 1RMH) [29]. The green labeled regions are flexible surface loops, showing large displacements in X-ray structures (large temperature factors) and NMR relaxation studies. A peptide substrate is shown as orange ball-and-stick model.

The peptidyl-prolyl *cis/trans *isomerase (PPIase) activity of cyclophilin A (CypA) has been investigated in detail for the link between protein dynamics and enzymatic catalysis, both by biochemical experiments and theoretical methods [10-12,15,16]. CypA is a ubiquitously expressed cytosolic protein, which was discovered as the major intracellular receptor protein for the immunosuppressive drug cyclosporin A [24,25]. CypA belongs to the cyclophilin class of enzymes, which are involved in many biological reactions including protein folding, intracellular protein transport and signaling [26,27]. CypA acts as a PPIase, catalyzing the isomerization of peptidyl-prolyl amide bonds that are N-terminal to proline residues in a wide variety of peptides and protein substrates (see Figure 2) [26,28]. Human CypA is a single peptide chain with 165 amino acids. Its molecular architecture consists of an eight-stranded anti-parallel β-barrel with hydrophobic residues forming a core at the center and the active-site located on one face of the molecule (see Figure 3) [29-31]. In addition to the β-strands and α-helices, there are several flexible surface loop regions as indicated by large temperature factors from X-ray crystallographic studies.

A number of factors make CypA an attractive system for investigating the link between internal protein dynamics and enzymatic activity; it is a small protein and does not require metal ions or cofactors for PPIase activity and it catalyzes peptide bond isomerization in a wide variety of substrates. Further, there is also biomedical interest in CypA; cyclophilins are of interest as drug targets because of their likely involvement in the broad spectrum, anti-infective activities of cyclosporin A and non-immunosuppressive derivatives thereof [32,33]. In addition, Gag-encoded capsid protein (CA) from human immunodeficiency virus type 1 (HIV-1), is a naturally occurring biologically relevant substrate for CypA [16]. The protein-protein complex between CypA and CA has been the subject of many experimental studies [16,34-37]. There is medical interest in CypA-CA complex, as incorporation of CypA is required for infectious activity of HIV-1 [38,39].

**Figure 4:**
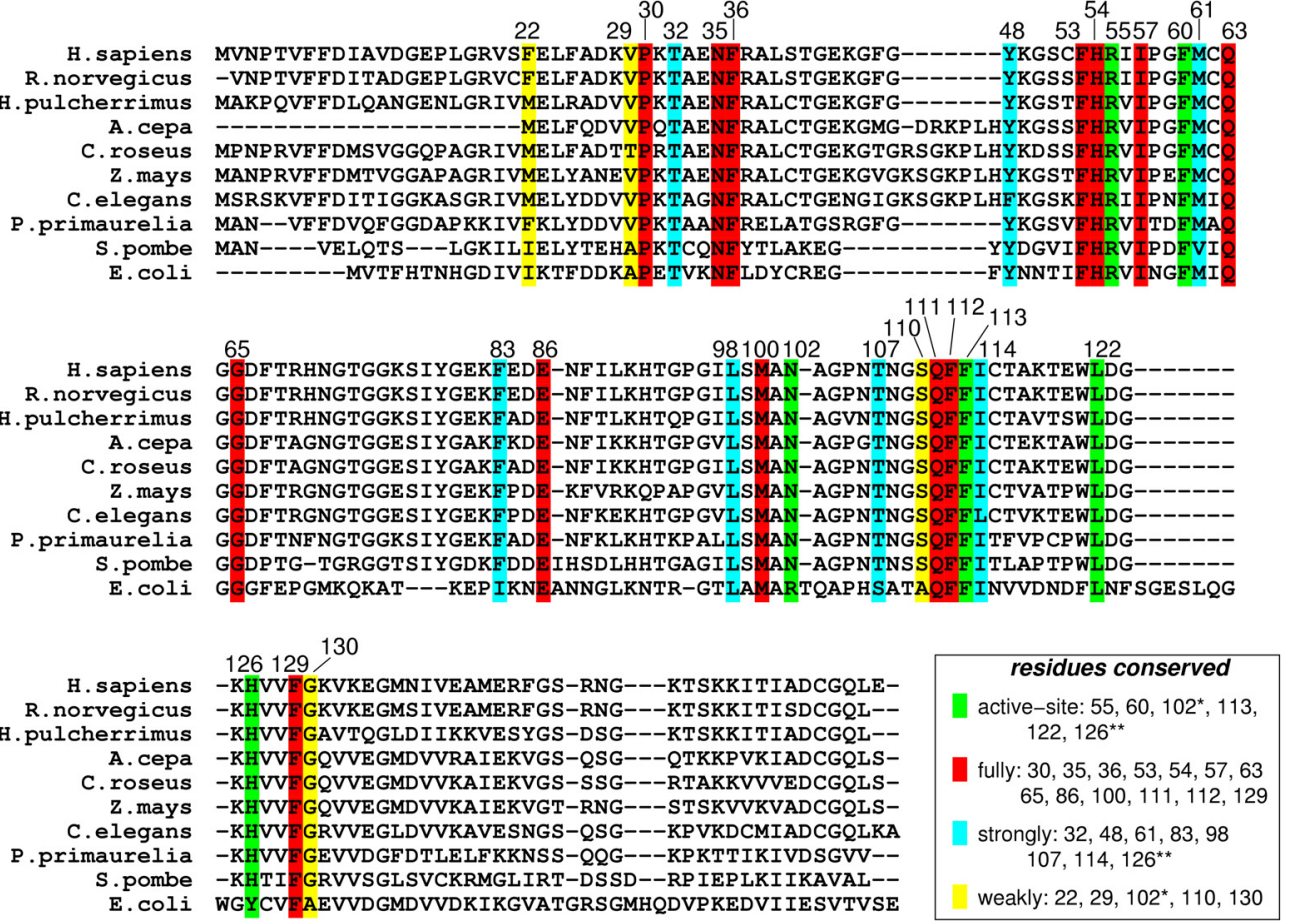
Genomic analysis for sequence conservation of CypA. Full analysis was performed on 50 sequences from species ranging from bacteria to human, results from 10 representative sequences are listed above. 17 of 165 amino acid residues in the human CypA sequence were found to be conserved in all 50 PPIases sequences examined: Pro30; Asn35; Phe36; Phe53; His54; Arg55; Ile57; Phe60; Gln63; Gly65; Glu86; Met100; Gln111; Phe112; Phe113; Leu122; and Phe129. Eight additional residues were found to be strongly conserved: Thr32; Tyr48; Met61; Phe83; Leu98; Thr107; Ile114 and His126. Five other residues were found to be weakly conserved: Phe22; Val29; Asn102; Ser110 and Gly130. Conserved active-site residues (fully conserved Arg55, Phe60, Leu122; strongly conserved His126**; and weakly conserved Asn102*) are shown with green background. Red background shows fully conserved residues distal to the active-site; residues with cyan background are strongly conserved; and residues with yellow background are weakly conserved.

Genomic analysis based on multiple sequence alignment has identified conserved residues in the CypA active-site and also distal to the active-site. This analysis was based on aligning 50 PPIase sequences from 25 diverse organisms, ranging from bacteria to human [10]. The results from this analysis are depicted in Figure 4. Detailed structural insights have indicated that the active-site of CypA shows conserved residues forming crucial hydrophobic and hydrophilic interactions with the substrate residues [see Figure 2(b)]. In addition, there are several conserved and semi-conserved residues that are more than 12 Å from the active-site. Until recently, the role of these distal residues in the enzyme function was not very well understood. As described below the dynamical motions of some of these residues have been found to play a role in catalysis.

NMR studies of CypA performed by Kern and coworkers, have suggested a link between internal protein dynamics and substrate isomerization step [15,16]. The studies were based on ^15^N spin relaxation investigations of small peptide substrate as well as two-dimensional (2D) ^1^H-^15^N heteronuclear exchange studies of the N-terminal of capsid protein (CA^N^) from HIV-1. In these studies, conformational fluctuations within the active-site of CypA were detected on the time-scale of the reaction (hundreds of microseconds) and the rates of conformational dynamics were found to be strongly correlated with the substrate isomerization step. Several active-site and surface loop regions showed motions only in the presence of substrate, these regions included the residues: Arg55, Lys82, Leu98, Ser99, Ala101, Asn102, Ala103, and Gly109. Based on these studies, the authors proposed a reaction mechanism for PPIase activity of CypA, where the isomerization step takes place with a rate constant of about 9000 s^-1^, and motions of the protein coincide with the rate of substrate turnover step. CypA residue Arg55 is a major contributor to catalysis [40], for which the observed changes in backbone conformation are likely to be coupled with motions of the catalytically essential side chain.

**Figure 5:**
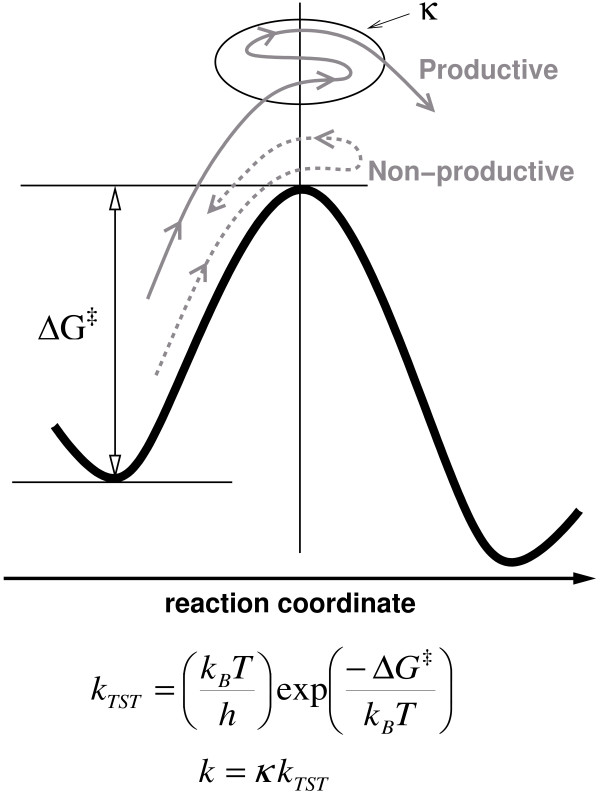
Schematic illustration of free energy profile for an enzymatic reaction. Protein dynamics can influence reaction rates in two possible ways; by altering height of the activation free energy barrier (ΔG^‡^) and transmission coefficient (κ). *k*_*B *_is the Boltzmann's constant, *T *is the temperature, *h *is the Planck's constant and *k*_*TST *_represents the transition state theory reaction rate.

Theoretical and computational modeling of the PPIase activity of CypA has provided novel insights into understanding the relationship between dynamical events in proteins and enzyme catalysis, including the mechanism of rate-enhancement achieved by enzymes [10-12]. Note, the time-scale of CypA reaction is hundreds of microseconds, which is beyond the reach of present day molecular dynamics simulations. Molecular dynamics, the commonly used computational technique, is limited to nanosecond time-scale (up to 100 nanoseconds at best) due to the limitation of available computing power. A different theoretical framework was, therefore, used for detailed computational modeling of the entire reaction pathway. This framework is described briefly below, more details are available in other reviews [9,41]. The theoretical investigations of enzyme catalysis are based on description of the reaction using transition state theory (TST) and generation of a free energy profile as a function of suitable reaction coordinate. In the TST framework, protein dynamics has been suggested to impact the reaction rate in two ways. Enzymes can either decrease the activation energy barrier (ΔG^‡^) for the reaction or alter the active-site conditions such that more reactive trajectories are converted to product successfully. Figure 5 shows the behavior of two trajectories, the first trajectory crosses the transition state (TS) barrier but is unsuccessful and returns to the reactant side. The second trajectory crosses the barrier several times before becoming productive. Transmission coefficient (κ) is a corrective pre-factor corresponding to the fraction of reactive trajectories that successfully cross the TS barrier and become productive. For CypA, the free energy profiles were generated using the amide bond dihedral angle of the peptide bond as reaction coordinate. Note, in the computational studies described here the unit of reaction coordinate is degrees (°). The free energy profiles were generated for isomerization of 3 small peptide substrates as well as the biologically relevant substrate CA^N^. The procedure for generation of these profiles requires multiple simulations of small sections along the reaction path by using molecular dynamics and umbrella sampling [42], and combining them to provide information regarding the time-scale of the reaction [43]. More details about the computational methods can be found in refs. [10] and [11]. In addition to obtaining the free energy profile this modeling procedure also sampled the enzyme-substrate conformations along the entire reaction pathway. These conformations have been used for detailed analysis of structural and dynamical changes during the enzyme reaction mechanism.

Structural analysis of the active-site along the course of reaction indicates the role of important hydrophilic (Arg55 and Asn102) and hydrophobic (Phe60, Phe113, Leu122 and His126) residues of CypA in stabilization of the substrate peptide. The target proline from substrate remains essentially fixed in the hydrophobic pocket formed by CypA residues, while the carbonyl oxygen atom of the preceding substrate residue rotates 180°. Quantum mechanical modeling of the active-site indicates single bond character for the peptide bond near the TS. The results from theoretical modeling were found to be in agreement with the reaction mechanism proposed on the basis of crystallographic studies [37]. This mechanism requires minimum deviation from the ground state crystal structure and displays single bond character for the peptide bond near TS. Dynamical fluctuations of the enzyme backbone in certain regions (CypA 101–104) were found to impact the nature of interactions between the enzyme and substrate, therefore, alter the nature of peptide bond during the course of reaction mechanism.

Computational modeling has identified a variety of internal protein dynamical events linked to CypA enzyme activity, ranging from femtosecond (10^-15 ^s) to microsecond and longer (> 10^-6 ^s) time-scales. On one side of this range there are fast motions, occurring at femtosecond-nanosecond time-scales, consisting of harmonic movements of bonds, angles and a few atoms. These motions are commonly referred to as vibrations. On the other side of this range there are concerted conformational fluctuations occurring on the microsecond (and longer) time-scale. These slower motions or conformational fluctuations, which have been previously referred to as *breathing motions*, span a large part of the protein. Normal mode analysis is a computational technique that has been commonly used to obtain information regarding the dynamical motions in proteins. This technique provides information about dynamics at several time-scales for a particular protein conformation (present at a local minimum). Normal mode analysis is not suitable for obtaining the slow protein motions occurring at the time-scale of the reaction due to the large changes in protein conformations involved. Another computational technique, known as quasi-harmonic analysis, can be used to calculate vibrational modes from a collection of conformations or system snapshots [44]. Quasi-harmonic analysis of CypA-substrate conformations along the entire reaction pathway provided protein vibrational modes representing conformational fluctuations at the time-scale of the reaction (microsecond-millisecond time-scale). These computed slow protein vibrational modes show concerted motions over a large region of the protein, the backbone in several regions of the protein and side-chains of the many residues (especially on the surface) show large displacements. In CypA, a subset of these modes was found to be coupled to the reaction; 3 protein vibrational modes with the largest coupling to the catalytic step show displacements in several conserved residues in the active-site as well as in other parts of the enzyme structure. Note, these conserved vibrational modes are different from random thermal fluctuations observed in the biomolecules.

**Figure 6:**
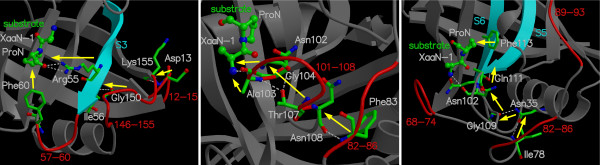
A network of coupled protein vibration promoting catalysis in cyclophilin A [10]. Alternate pathways by which protein dynamics impacts the enzyme reaction are depicted based on 3 protein vibrational modes coupled to the reaction. Loops colored in red and residues indicated by ball-and-stick show largest displacements in vibrational modes coupled to the substrate turnover step. The yellow arrows represent the network pathway from outside of the enzyme to the active-site. *Reprinted with permission from Agarwal et al., Biochemistry (2004) ****43***, *10605–10618*. Copyright 2004 American Chemical Society.

The detailed characterization of internal protein dynamics events linked to enzyme catalysis in CypA has lead to the discovery of a network of protein vibrations (see Figure 6). This network plays an important role in promoting the isomerization reaction [10-12]. The discovery of this network is based on identification of 3 protein vibrations on the time-scale of the reaction, investigation of the dynamical flexibility of the CypA backbone, monitoring the conserved residues and interactions over the course of enzyme reaction. Dynamical cross-correlation analysis of enzyme parts indicated that several surface loops (distant to each other in sequence) show highly correlated motions during the course of the reaction. These correlated motions form the network of vibrations through a series of interactions, as shown by yellow arrows in Figure 6. Note that this network extends from the surface regions of the enzyme all the way to the active-site, through interconnection of conserved residues and interactions. The vibrations in this network are transmitted to the active-site, where dynamical motions alter the crucial hydrophobic and hydrophilic interactions between enzyme and substrate. As noted above these interactions play a critical role in the reaction mechanism by controlling the nature of the peptide bond, as well as in rotation of the carbonyl oxygen atom from the residue preceding the target proline of the substrate. Evidence for the existence of this network comes from previous NMR studies, where motions have been detected in network residues only during the substrate turnover [15]. Further, the flexibility of the network residues is confirmed by observation of large temperature factors in X-ray studies [29-31,37]. More recently, new investigations performed by Kern and coworkers, using NMR studies have confirmed the presence of this network in CypA [45]. Moreover, NMR investigations conducted by Blackledge and coworkers have also observed the role of protein motions in transfer of information between β-strands [46]. These recent findings have illustrated the role of coupled networks in propagation of local changes over large distances in protein structure.

**Figure 7:**
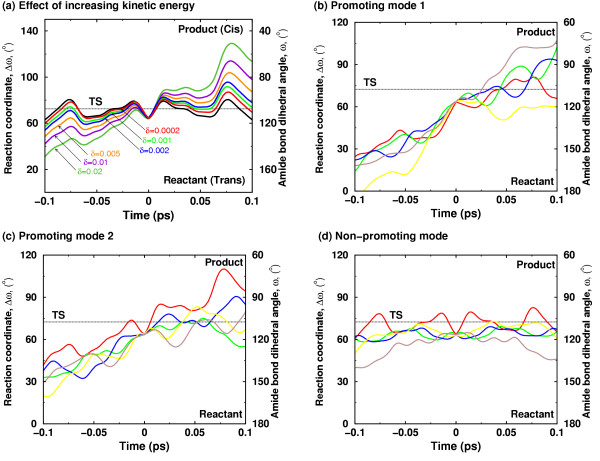
Effect of additional kinetic energy in selective protein vibrational modes (a) Increased amount of kinetic energy in a mode coupled to the enzyme reaction allows the trajectory to cross the barrier successfully from the reactant side to the product side. The solid black curve represents the native trajectory (no additional kinetic energy), and δ indicates the fraction of system kinetic energy added to the protein vibrational mode. (b)-(d) representative trajectories from simulations with increased kinetic energy in network protein vibrational modes and a non promoting mode. 2% of system kinetic energy was added to protein vibrations mode. Five representative trajectories from each mode are shown in different colors. Not all protein vibrational modes show increased barrier recrossing; much less effect on the barrier crossing is seen in a mode not coupled to the reaction [12]. *Reprinted with permission from Agarwal et al., J. Am. Chem. Soc. (2005) ****127***, *15248–15256*. Copyright (2005) American Chemical Society.

The discovered network of protein vibrations has a promoting effect on the CypA enzyme activity, and is therefore, a factor contributing to rate-enhancement. Certain protein vibrational modes alter the reaction by changing the active-site environment such that more reaction trajectories cross to the product side. A new theoretical technique has been designed and was used to investigate impact of reaction coupled vibrational modes on the reaction mechanism [12]. This technique allows addition of kinetic energy to a selective vibrational mode and observing the dynamical behavior of the trajectory (see Figure 7). Figure 7(a) shows the change in behavior of trajectories with increasing amount of kinetic energy present in a protein vibrational mode. The result from further investigations show that the presence of energy in certain reaction coupled promoting modes causes the reaction trajectories to overcome the activation energy barrier quickly and more effectively [see Figure 7(b)–(c)]. Note that not all modes promote the reaction, as indicated by a non-promoting mode [that is a mode which is not coupled to the reaction; see Figure 7(d)]. Also, the theoretical investigations were performed by adding varying amounts of energy to see the effect of these vibrations in a short simulation (picosecond time-scale). The trend indicates that smaller amounts of kinetic energy present in these modes, which is expected to be present in real system, promotes the reaction at longer time-scales (hundreds of microseconds). The biophysical role of the discovered network in the enzyme reaction can be understood by observing changes that are introduced in the active-site by reaction promoting vibrations. Detailed analysis indicates that the dynamical behavior of reaction trajectories is correlated with the fluctuations in the enzyme-substrate interactions as a result of increased energy in the protein vibrational mode. Rate-enhancing modes impact the key active-site interactions to make the reaction proceed from reactant side to the product side. An interesting observation, from this analysis, is that the maximum enzyme stabilization occurs close to the TS (consistent with the TS stabilization theory for enzyme catalysis). The role of the reaction promoting vibrations could, therefore, be interpreted as internal protein dynamical events that facilitate in the stabilization of the TS.

**Figure 8:**
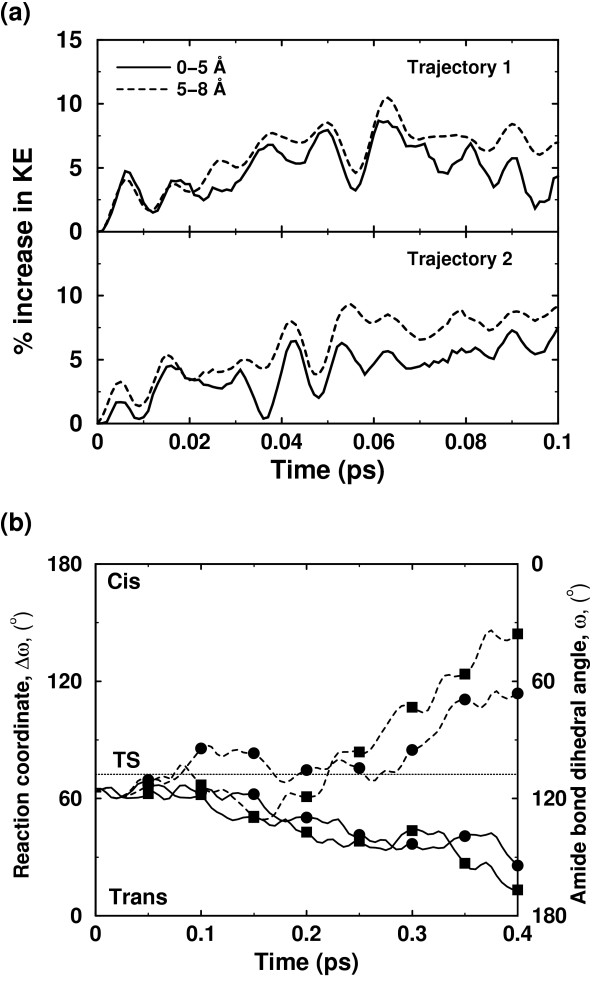
Effect of additional kinetic energy in first solvation shell of an enzyme. (a) Kinetic energy is transferred from the solvent to the protein residues, as indicated by increasing energy in the protein regions (up to 5 Å from protein surface, and between 5 Å and 8 Å from the surface) (b) Two otherwise non-productive regular trajectories (solid lines) become productive (broken lines) due to transfer of energy from the solvent to residues forming parts of the protein vibrations network. The corresponding trajectories are indicated by squares and circles [12]. *Reprinted with permission from Agarwal et al., J. Am. Chem. Soc. (2005) ****127***, *15248–15256*. Copyright (2005) American Chemical Society.

Solvent surrounding the enzyme also plays a role in the enzyme reaction. In many enzyme reactions, hydrolysis of small molecules provides the energy for overcoming the activation energy barrier; however, in other cases the required energy is provided by the thermodynamical fluctuations of the solvent. The fluctuations in the hydration-shell and bulk-solvent surrounding the enzyme are correlated with the internal protein motions. Detailed characterization of the flexible surface loop regions indicates that the side-chains of several surface residues extend into the solvent and the motion of these residues is coupled to the motion of surrounding solvent molecules. CypA investigations indicated the presence of vibrations (on picosecond) time-scale in several crucial surface residues, which are present in the loop regions showing large displacements in reaction promoting vibrational modes. Previous theoretical investigations have also shown that the transition in internal motion of proteins can be driven by the temperature of the solvent [47,48]. In CypA, computational modeling has shown transfer of energy from solvent to the external regions of the enzyme. This energy transfer changes the behavior of reaction trajectories, through the network of protein vibrations, to promote catalysis (see Figure 8).

An interesting outcome of detailed characterization of the network of protein vibrations in CypA is the insight into the conservation of protein residues. The genomic analysis for sequence conservation reveals that there are several residues which are conserved due to their dynamical role in catalysis. Active-site residues, which are key players in the catalytic step, are often conserved in different species. In addition to the active-site residues (Arg55, Phe60, Asn102 and Ala103), which directly participate in the catalytic step, there are several distal residues (including Pro30, Asn35, Phe36, Phe83, Glu86), which are also conserved. Note, that some of these residues are more than 17 Å from the active-site. Results from detailed structural analysis (summarized in Table 1) indicate that these and other distal residues form crucial points and hydrogen bonds in the discovered network and, therefore, are conserved in species ranging from bacteria to human. This finding also has interesting implications on the understanding of the secondary and tertiary protein structure. The discovered network promoting catalysis may provide some insights into the conservation of protein "folds"; enzymes catalyzing similar reactions often belong to the same fold family, and enzymes catalyzing mechanistically similar reactions belong the same protein super-family [49,50].

**Table 1:** Conservation of network hydrogen bonds in cyclophilin structures from various species. 3-dimensional structures were aligned using secondary structure elements and equivalent hydrogen bonds were selected based on sequence and structural similarities. Hydrogen bond lengths are in given Å and PDB codes are given in parenthesis [10]. *Reprinted with permission from Agarwal et al., Biochemistry (2004) ****43***, *10605–10618*. Copyright (2004) American Chemical Society.

CypA (1AWQ/2CYH/1RMH average)	Asp13N-Lys155O	Asn35N_δ2_-Gly109O	Ile56N-Gly150O	Ala101N-Gln111O	Phe83N-Asn108O
	2.89	3.01	2.96	3.04	2.87
Human Cyclophilin B	Gly21N-Asp164O	Asn43N_δ2_-Gly117O	Val64N-Asp159O	Ala109N-Gln119O	Phe91N-Asn116O
(1CYN)	2.91	3.06	3.00	3.05	2.75
*B. malayi*	Asp16N-Asp167O	Asn38N_δ2_-Gly120O	Val67N-Asn162O	Ala112N-Gln122O	Phe94N-Asn119O
(1A33)	2.89	2.92	3.05	3.06	2.85
*C. elegans*	Gly13N-Asp162O	Asn35N_δ2_-Gly116O	Ile63N-Gly157O	Ala108N-Gln118O	Phe90N-Asn115O
Cyclophilin 3 (1DYW)	2.85	3.09	2.96	2.98	2.94
*C. elegans*	Gly37N-Asp180O	Asn59N_δ2_-Gly133O	Val80N-Asp175O	Ala125N-Gln135O	Phe107N-Asn132O
Cyclophilin 5 (1H0P)	2.78	2.91	2.85	3.09	2.93
*B. taurus*	Gly25N-Leu175O	Asn47N_δ2_-Gly129O	Ile76N-Glu170O	Ala121N-Gln131O	Phe103N-Asn128O
PPIase (1IHG)	2.98	3.21	2.84	3.07	2.84
*P. falciparum*	Asp14N-Ser162O	Asn36N_δ2_-Ser116O	Ile63N-Gly157O	Ala108N-Gln118O	Phe90N-Asn115O
Cyclophilin (1QNG)	2.74	2.88	2.93	3.05	2.80
*E. coli*	Asn7N-Ile156O	Asn26N_δ2_-Thr95O	Val44N-Asp149O	Ala86N-Gln97O	Ile68N-Ala94O
PPIase (2NUL)	2.90	2.67	2.97	2.88	2.76

### Other enzymes: dihydrofolate reductase and liver alcohol dehydrogenase

Experimental and computational investigations have revealed the impact of protein dynamics on catalysis in other enzyme systems. Experimental and computational studies of the enzyme dihydrofolate reductase (DHFR) have indicated a link between protein dynamical events and the substrate turnover step of hydride transfer. X-ray crystallography has demonstrated changes in orientation of surface loops along different sub-states along the reaction pathway [51]. Similarly, the surface loop conformations have been linked to the catalytic step by NMR studies [20]. Theoretical and computational studies using hybrid quantum-mechanical and molecular mechanics (QM/MM) methodology have discovered a network of coupled promoting motions [8,52,53]. Similar to the network of protein vibrations in CypA described above, the network in DHFR is also formed by interconnection of residues and crucial interactions ranging from surface regions all the way to active-site. Changes in hydrogen-bonds and crucial interactions along the reaction profile have been observed similar to those present during catalysis by CypA. An important discovery by the computational methods was the identification of the residue Ile14 as a dynamical contributor to catalysis. Recently, the importance of this residue in the catalytic step has been confirmed by NMR studies [54]. The presence of this DHFR network has been confirmed by investigations from several research groups [55,56]. Investigations of DHFR have provided evidence that changing the enzyme structure leads to changed dynamics and, therefore, change in function [21,53,57]. Mutation of a single surface residue, more than 12 Å away from active site, changes the dynamics and leads to a rate reduction by a factor of 163.

Liver alcohol dehydrogenase (LADH) is another enzyme where dynamical motions of the protein residues have been linked to the catalytic step. Detailed biochemical and computational studies have identified conserved active-site residue Val203, whose motion are a key player in altering the active-site chemical environment to promote the reaction [58-63].

## Conclusion

In this review, we have presented recent developments that continue to support an emerging integrated view of protein structure, dynamics and function such as enzyme catalysis. The success of microbial cell factories depends on optimal performance of molecular machines inside the cell. Enzymes perform their function with remarkable efficiency, as they increase the reaction rate by many orders of magnitude. Until recently, enzymes (and proteins in general) were considered static assemblies; however, recent investigations continue to provide evidence which indicate that enzymes are dynamically active assemblies. Detailed experimental and theoretical/computational investigations of enzyme CypA suggest that the internal protein motions are a designed part of the protein structure and contribute to its function of catalyzing peptidyl-prolyl *cis/trans *isomerization. Supporting evidence from other systems (DHFR and LADH) indicates that the interconnection between structure, dynamics and function is present in other enzymes as well.

**Figure 9:**
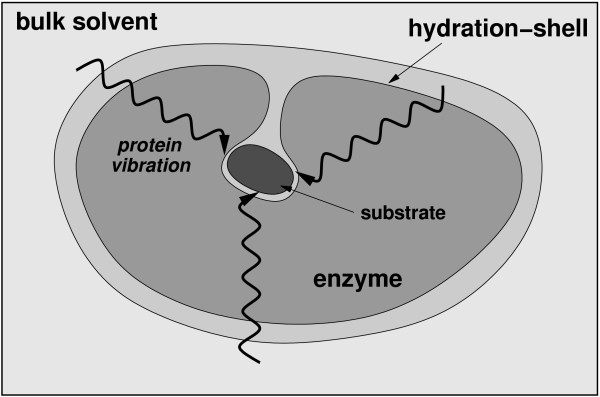
**A schematic representation of the integrated view of enzyme structure, dynamics and function**. Enzyme structure and internal protein dynamics events play a role in the catalytic step. Conserved residues from the surface to the active-site participate in network of protein motions or vibrations that promotes catalysis. The surface residues are coupled to the thermo-dynamical fluctuations of the solvent, and possibly play a role in transfer of energy from solvent to the protein.

The overall emerging picture of protein dynamics, solvent fluctuations and enzyme function based on recent insights is depicted in Figure 9. Along with structural interactions, internal motions at fast time-scales control the chemical environment of the active-site favoring the catalytic step to proceed to the product state. The thermodynamical fluctuations of the hydration-shell and bulk solvent provide energy to overcome the activation energy barrier (in cases where no other source of energy is available). The flexible surface loop regions of the enzyme show dynamical coupling with the solvent. This dynamical coupling allows the transfer of energy from the solvent to the surface regions of the enzyme. This energy is eventually transferred to the active-site through networks of motions or vibrations. The slower conformational fluctuations in the networks (at time-scale of the reaction) alter the enzyme-substrate interactions such that more reaction trajectories cross TS barrier to reach the product state successfully.

The integrated view is supported by evidence from investigations of many other proteins and enzymes as well [64-66]. Sequence analysis with thermodynamic mapping have indicated long range energetic coupling in proteins [67]; slow conformational fluctuations could possibly be the mechanism of energy transfer over long ranges in protein structure and, therefore, provide insights into understanding allosteric effects. Simulations have already revealed that energy can be transferred between specific vibrational modes in a protein [68,69]. It is also interesting to note that designing active-site mimics of the enzymes is difficult and change in enzyme structure far away from the active-site leads to slow or inactive enzymes. The integrated view offers a possible explanation, as the distal regions of the enzyme contribute to catalysis through dynamical coupling with the solvent and by transferring the required energy to the active-site. Therefore, this integrated view has wide implications in enzyme chemistry, protein engineering and drug design. Manipulation of enzyme catalyzed reactions may be possible; for example, laser pulse has already been used to initiate an enzyme reaction involving thermally excited protein dynamics (molecular motions on picosecond time-scale) [70]. On the basis of better understanding of enzyme structure, dynamics and function it may be possible to design more efficient enzymes or enzymes with novel functionalities. Further, the understanding of allosteric and cooperative effects could help in designing better and novel drugs.

## List of abbreviations

κ, transmission coefficient

ΔG^‡^, activation energy barrier (energy difference between reactant and the activated state)

CA, capsid protein from HIV-1

CA^N^, N-terminal of capsid protein

CypA, cyclophilin A

DHFR, dihydrofolate reductase

HIV-1, human immunodeficiency virus type 1

LADH, liver alcohol dehydrogenase

NMR, nuclear magnetic resonance

PPIase, peptidyl-prolyl *cis/trans *isomerase

TS, transition state

TST, transition state theory

## Authors' contributions

PKA drafted and revised the manuscript.
